# Transnational intergenerational relationships and doing transcultural family through mandarin learning: an ethnographic glance at Chinese dynamics in four intercultural families in Germany

**DOI:** 10.3389/fsoc.2024.1498744

**Published:** 2024-12-03

**Authors:** Jiayin Li-Gottwald

**Affiliations:** Faculty of Humanities and Social Sciences, Helmut Schmidt University, Hamburg, Germany

**Keywords:** third space, transnational intergenerational relations, doing transcultural families, Mandarin Chinese, intercultural families

## Abstract

This article explores the micro dimension within the field of intercultural family studies. The ethnographic study focuses on Chinese elements in transcultural upbringing practices within families with Chinese mothers and German fathers raising their children in Germany. It builds on the notion of family figuration practice in transnational grandparents-grandchildren relations, particularly Confucian-based transnational grandparenting, and the development of a ‘third space’ for transcultural family upbringing within intercultural families. Additionally, it incorporates the concepts of discourse perspective of culture and assemblage to explore how Chinese culture and identity converge in a transcultural family upbringing context. The findings show that families have varied experiences with transnational intergenerational communication influenced by their life stage, language abilities, and socio-economic factors.

## 1 Introduction

This study explores transnational intergenerational relations and transcultural upbringing in families with Chinese mothers and German fathers raising their children in Germany. The paper is a component of a wider ethnographic project that investigates different facets of intercultural families. Although the current article specifically addresses Chinese dynamics in the intercultural family upbringing process, it is crucial to note that this topic is only one of many topics within the overall framework of the broader ethnographic research.

The paper complements existing transnational studies on human mobility between host and sending countries, which primarily focus on the overall process of human transportation within these corridors (Faist, 1999; Pries, 2001; Levitt and Jaworsky, 2007; Vertovec, 2009). Following transnational family intergenerational relation studies with the focus on grandparenting (Moran-Taylor, 2008; Bastia, 2009; Nedelcu and Wyss, 2020), this article showcases a distinctive shift of transnational grandparents/children relation in intercultural families, focusing on intergenerational age-specific and linguistic obstacles. In addition, this paper adopts Crippen and Brew’s (2007) definition of transcultural family as ‘the new family culture that is created when the cultures of two people from different backgrounds intersect to form a new culture’ (p. 107), showcasing a distinctive focus on specific moments in transcultural ‘doing family/ies’ (Morgan, 1996, 2011; Finch, 2007; Higgins and Hamilton, 2014; Jurczyk et al., 2014) through Chinese Mandarin language learning. Based on the notion of the discourse perspective of culture (Kramsch, 1998, 2014; Holliday, 2018), transculturalism (Epstein, 2009; Baker, 2024), and the assemblage of linguistic and non-linguistic interactions (Pennycook, 2019), the study underscores the significance of object assemblage, human interactions, and cultural interpretations within particular domestic settings, exploring the development of the transcultural family through the dynamics of transnational intergenerational family relations and everyday transcultural child upbringing. The central theme of the article revolves around an in-depth analysis of how four families living in Germany navigate these multifaceted aspects. The main body addresses three research questions: (1) How are transnational intergenerational family relations with grandparents in China manifested in everyday intercultural family life? (2) How is transcultural upbringing manifested in everyday intercultural family life through Mandarin learning in Germany? (3) What are the emerging discourses around these upbringing practices and what are the interconnectedness? The findings suggest that various families have different experiences regarding transnational intergenerational communication based on life stage, linguistic capability, and socio-economic backgrounds. Additionally, the learning of the Mandarin language has become a crucial process in the construction of their ‘doing transcultural family’. Data for this article are derived from ethnographic research, specifically participant observation and qualitative semi-structured interviews conducted between October 2022 and Oct 2023, focusing on fieldnotes (15 pieces), interviews (5 pieces) and photographs (2 pieces). The analysis of the data utilized content coding to uncover central themes in the field notes and interactional data, such as video and audio clips. This approach enabled me to draw meaningful insights that guided the formulation of additional questions for future interviews. Learning Mandarin is crucial for establishing and nurturing transcultural family connections, as well as for sustaining or weaken the intergenerational transnational relationships.

## 2 Studies of transnational grandparenting

Research has illustrated how migrants^1^ actively construct transnational social fields, carve out transnational spaces, and cultivate transnational belongings, providing compelling examples of migrants simultaneously sustaining connections with their countries of origin, while forging new relationships and cultivating a sense of belonging (Faist, 1999; Pries, 2001; Levitt and Jaworsky, 2007). Within the broader framework of transnational studies, transnational family relation studies concentrate on how global movements and networks shape family structures and practices (Bryceson and Vuorela, 2002; Baldassar et al., 2007, 2014). One emerging topic is transnational grandparenting revealing grandparents’ roles in the transnational circulation of care in modern transnational family transformations (Parrenas, 2005; Moran-Taylor, 2008; Bastia, 2009), and the transnational ‘doing family’ practices (Wyss and Nedelcu, 2018).

With a specific focus on Chinese migrant families, numerous studies have shed light on the phenomenon of grandparenting within transnational Chinese families residing in the United States, Europe, and China. One notable study by Lamas-Abraira (2021) uncovered the practice of sending children from Spain back to their parents’ family of origin in China, where they were raised by their grandparents. This transnational arrangement showcases how care responsibilities are shared and circulated across borders. Similarly, Wang and Schwartz (2016) conducted research on Chinese grandparents who assume childcare duties while residing in Paris with their children. This study highlights the role played by grandparents in transnational Chinese families, emphasizing their involvement in caregiving and their presence in foreign contexts. Xie and Xia (2011) closely examine grandparenting within Chinese immigrant families in the United States, exploring the continuity of grandparenting culture in a transnational context. The authors state that Confucianism, emphasizing family structures and intergenerational relations, shapes the ‘mutual aid model’ of Chinese families (Sun, 2002). This model, exemplified by grandparents caring for their grandchildren and aiding for the young families, continues in Chinese immigrant families in the United States. While previous empirical studies primarily address Chinese transnational families with ethnically homogamous couples (Eeckhaut et al., 2011), there is limited research on intergenerational relations with a focus on grandparenting within intercultural families involving a Chinese partner. One important theme of this article is how Chinese Confucian-based grandparenting functions in culturally mixed transnational families.

## 3 Culture, transcultural family and multilingual upbringing

Matsumoto and Juang (2008) define culture as a shared system of meaning, including values and beliefs, among members of a cultural group who have experienced the same process of cultural socialization (Umaña-Taylor and Fine, 2004). Taking a more discursive approach, Kramsch (1998, 2014) describes culture as belonging to a discourse community with shared social spaces, history, and collective imaginations. Members of this community, even after leaving it, often retain a common system of standards for perception, belief, evaluation, and action. These standards are typically referred to as their ‘culture’. In this study, ‘intercultural families’ refers to couples with differing cultural socialization, historical experiences and linguistic backgrounds forming a close and intimate relationship, raising children together. Specifically, the paper discusses four families, each comprising a mother from China and a German father, who have children together in Germany.

According to Crippen and Brew (2007, 2014), the development of a family is influenced primarily by two factors: the identity and culture each partner brings from their own background, and the identity and culture the couple creates together through marriage and parenting. Recent studies show that cultural differences are particularly pronounced in migrant families and intercultural couples, who often find themselves navigating the crossroads of differing family values, language ideologies, norms of interaction, and identities (Song, 2010). In particular, intercultural families face unique challenges in raising children because each parent contributes a distinct cultural heritage and identity to the family dynamic. This negotiation process often results in the emergence of a ‘third space’ (Bhabha, 1994), where a ‘transcultural family’ system develops (Crippen and Brew, 2007), blending elements from both parents’ backgrounds. The concept of the ‘third space’, initially proposed in Bhabha’s (1994) studies, emerges from the blending of different cultural elements within a liminal or in-between space where the ‘cutting edge of translation and negotiations’ (p.56) takes place, conceptualizing coloniality and postcoloniality through hybridity, liminality, and in-betweenness to transcend the polarity of self and other, East and West, and envision a hybrid space where cultures interact and overlap (Bhandari, 2020).

This notion of ‘third space’ offers a framework for understanding the blending and negotiation of cultural elements within a liminal zone, which aligns with transcultural studies’ focus on the fluid and dynamic nature of cultural exchange and transformation beyond rigid boundaries. Taking an everyday discourse perspective, culture is understood as discursively produced in everyday life (Silverstein, 2013). Transculturalism refers to the emergence of new cultural forms as a consequence of intercultural contact in culturally diverse populations (Cuccioletta, 2002). Thus, in a transcultural family, the integration of diverse cultural elements goes beyond simply maintaining separate cultural identities. Instead, it involves creating a new, shared family identity that draws from and incorporates aspects of each parent’s cultural heritage, and is a lifelong learning process (Vauclair et al., 2024) for the intercultural families. This process transforms the family dynamic into something greater than the sum of its parts, creating a unique cultural milieu that transcends the original cultures of each parent, ultimately leading to the development of a ‘transcultural family system’ (Crippen and Brew, 2007).

Among other factors in the process of constructing a transnational family system, recent research shows that multilingual and intercultural practices are significant for many transcultural and transnational families (Lanza and Curdt-Christiansen, 2018; Lanza and Wei, 2016). Thus, multilingual child-rearing has been one of the most important aspects in the development of transcultural families. These studies point out that Language ideology and language choices for the children which reflect the parents’ views about the socio-cultural values of different languages are pivotal measures of transcultural family upbringing. In intercultural families, the socio-cultural values associated with different languages may vary significantly across generations and can be motivated by personal, social, cultural and political factors (Lanza and Curdt-Christiansen, 2018). Consequently, multilingual child-rearing can create a third space for transcultural family practices, fostering negotiation, contestation, and evolution in transnational intergenerational relations and family dynamics.

Thus, this paper explores how intercultural couples adjust to and reconcile differences in multilingual parenting while forming a transcultural family, and how these practices influence relationships between transnational grandparents and their grandchildren.

This study aims to adopt a broader perspective on the construction of everyday moments in intercultural family upbringing by examining transcultural family systems through the process of their children’s Chinese-Mandarin learning. It investigates how ‘diverse trajectories of people, semiotic resources, and objects converge at specific moments and places’ (Pennycook, 2019, p. 86), and how these varied resources collaboratively contribute to the construction of meaning (Canagarajah, 2017). It seeks to transcend the confines of locality in transnational research and endeavors to shed light on the spatial, cultural, and social transcultural mobility practiced within a particular moment and space in the participant families. In addition, the emphasis on ‘transcultural family’ shifts the focus from nationalities and geographic dispersion to cultural diversity and transformation, highlighting the blending and adaptation of diverse cultural backgrounds within the family.

## 4 Research design and methodology

This research aims to explore everyday family upbringing practices exhibited by participatory families. The focus of this investigation is directed toward the manifestation of transcultural ‘doing family’ through multilingual child upbringing practices and the transnational grandparenting within ‘transnational marriage’ (Charsley, 2005) families residing in Germany. As mentioned earlier, the data presented in this article are derived from participant observation and qualitative semi-structured interviews. The ethnographic approach employed in this study offers valuable insights into family life (Ekström, 2004), enabling a profound exploration of the complex dynamics of transnational intergeneration communication and transcultural family practices within intercultural families.

Some data from four families residing in Germany will be presented in this article, three families were composed of ‘heterogamous couples,’ namely Chinese wives and German husbands, and their children whereas one family contains a single Chinese mother with her daughter residing in Germany. However, in the latter case, the Chinese woman shared a similar path of ‘transnational marriage’ in which she married an Italian man in Italy, later got divorced, and made her way to Germany with her daughter.

Three families were chosen from my close circle of friends whom I have known since my arrival in Germany 15 years ago. One of the wives I met during a German integration course that we both attended, while I met the other wife through my daughter’s piano lessons. For the purpose of this discussion, I will refer to them as the Bey’s and Wey’s families. The third family was introduced to me by an acquaintance. This family which I refer as Lin’s family, consists of a mother and a teenage daughter. As I conducted field visits, I noticed a distinct characteristic in the process of arranging these visits. In contrast to some ethnographic studies of transnational families, where arranging appointments required careful preparation and persistence, sometimes taking months (Li-Gottwald et al., 2024), the field visits in this study were often spontaneous and immediate. This spontaneity was facilitated by our regular and intensive mobile communications, which were based on the close relationships I had established with the families for years prior to this study. Although friendships and close relationships in ethnographic research can enhance the research experience, benefiting all parties involved (Huisman, 2008), it is important to notice special ethical concerns need to be paid in ethnographic research on close circles (Hall, 2009; Jurczyk et al., 2014). Ethical considerations can encompass the importance of obtaining informed consent, safeguarding privacy, maintaining confidentiality, acknowledging power dynamics within the relationships, and practicing self-reflexivity (Irwin, 2006; Davies, 2008; Huisman, 2008; O’Reilly, 2009). In the context of doing ethnography in intimate social circles, confidentiality, disengagement, and power dynamics are argued as the most prominent dilemmas (Lupton, 2012; Hall, 2014).

## 5 Analysis and discussion

### 5.1 Four families

One of the original methodologies employed in this study was to observe and document the activities of families during their everyday life as well as the Christmas, New Year’s Eve, and Chinese Lunar Festival periods. These occasions often serve to strengthen familial bonds and contribute to the overall well-being of family life. They each bring unique socio-cultural backgrounds and migrant trajectories. Pseudonyms are used for all the participants and their family members.

### 5.2 Bey’s family: the narrative

Bey migrated to Germany from Shanghai in 2007 under the family reunion program to join her then-husband. Originally born in a city in Jiangsu province, Bey pursued her studies in Shanghai. After completing her education at a renowned university in Shanghai, she secured a position at a commercial bank in the city, where she encountered her future spouse. Following her husband’s path, Bey relocated to a major city in Germany, where she has resided. However, her marriage proved short-lived upon her arrival in Germany. Bey shared with me that she felt like a caged bird. Toward the end of their relationship, she was confined against her will. Eventually, Bey made the decision to leave and sought refuge in a women’s shelter. With no knowledge of the German language and lacking any friends or relatives for support, she faced challenges in finding employment.

After transitioning from the women’s shelter to a rented room, Bey enrolled herself in a German language school, where I became acquainted with her life. Her German language skills rapidly improved, and by the end of the term, she had successfully passed her B1 language proficiency exam. Notably, Bey also possessed excellent English language skills and actively participated in various social activities. I vividly recall her taking me to numerous social events where English served as the primary means of communication. Over the course of two to three years, Bey managed to secure a more suitable apartment and eventually met her current husband, a young German man. Together, they have two sons. Throughout the fieldwork period, the couple had been together for over 10 years. Bey’s husband, Fin, is a local German and works as a chief financial officer, with a demanding schedule of over 50 h per week that often keeps him away from home. As a result, Bey made the decision to become a full-time homemaker, devoting herself to caring for their two young boys, aged 5 and 9. The family resides in a three-bedroom apartment located in a prominent area of the city, known for its excellent schools and childcare centers. Additionally, they own a holiday apartment on a coastal island in China, where they spend time together during family vacations.

Bey’s husband earns a very comfortable income, and although Bey is as a highly skilled and well-educated Chinese woman who could easily find employment, she has the choice to concentrate on the household and raising the children. Bey’s experience as a migrant aligns participants portrayed in Simeng’s (2020) study on Chinese migrants residing in Paris. Individuals born in the 1970–1980s who possessed Bachelor’s or Master’s degrees before migrating to Europe. They relocated to Europe with the aim of pursuing their career and establishing a family. In a similar vein, Bey’s personal journey unfolded as she first encountered her former husband in the vibrant cityscape of Shanghai, China, and subsequently embarked on a transformative voyage as a love migrant to Germany.

### 5.3 Wey’s family^2^

Wey’s trajectory as a migrant aligns with group of participants described in Wang (2021) study of Chinese migrants to Europe born between 1982 and 1990, who pursued higher education in Europe and later married local citizens. Wey arrived in Germany at the age of 19, following her graduation from a prestigious music high school in Shanghai, China. She was raised in an affluent family in Shanghai, where her father is a successful business owner, and her mother holds a senior nursing position at a renowned hospital. Wey’s upbringing revolved around her musical talents, as she received private singing and piano lessons from the age of 8.

With her parents’ support, Wey embarked on her classical music educational journey in Germany, initially dedicating a year to mastering the German language. Subsequently, she secured admission as an undergraduate student at a distinguished local music college, where she later pursued her Master’s degree in music education. I had the opportunity to meet Wey during her young adult years when she gave piano lessons to my daughter. Wey did not ask for any financial compensation for her teaching services; instead, she used the opportunity to gain valuable experience for her future career in music education. It was during her time at university that she met her husband, Ben, who shared the same dormitory. Ben, a gentle and courteous individual, had relocated from Frankfurt to the city where Wey is.

Upon completing their studies, Wey embarked on music teaching career, working for various schools and different projects, while Ben found employment as a computer programmer in an international company. Before long, they exchanged vows and acquired a residence in an exclusive area of the city based on financial support provided by Wey’s parents in Shanghai. Wey told me that her parents had sold one of their numerous apartments in Shanghai to fund the purchase. The couple had a two-year-old daughter. Wey’s parents frequently visited them in Germany, traveling from Shanghai, while Wey, Ben, and their young daughter often embarked on weekend trips to Ben’s parents’ home in Frankfurt. Wey exemplifies the experiences of Chinese international students in Germany hailing from affluent urban families in China since the turn of the millennium. Her narrative in Germany falls into the category of proactive young students who ventured to Germany for pre-college and undergraduate education.

### 5.4 Mey’s family

I first encountered Mey during my second year of residence in Germany at a German language school, and our friendship has endured ever since. As a love migrant or family migrant, Mey embarked on her journey to Germany to unite with her then-husband. Hailing from a Chinese farmer family in a small village, Mey relocated to the rapidly developing city of Shenzhen in her early twenties. Despite not having had the opportunity to pursue higher education in China, she displayed determination by attending evening classes in Shenzhen to acquire English language skills and sales expertise. Leveraging her linguistic talents, she successfully honed both competencies and secured a position as an international sales assistant in a Japanese company, where she subsequently encountered her future German spouse at a professional conference^3^.

Mey’s love blossomed rapidly, and following her German partner’s departure from China to Germany, she eagerly reunited with him in Europe. The couple became engaged at the iconic Eiffel Tower in Paris. Her husband, a CEO of a logistics company, and Mey settled in a suburban area of a major German city, constructing a home together. However, their marriage proved short-lived. Subsequently, Mey entered into a second marriage with a Northern German man employed in the finance department of the German Government. At the time of the fieldwork, they were married and had a teenage daughter. Despite her husband Den’s insistence that Mey reduces her workload, she works for two part-time positions. One is working as a caretaker in a local German school, while the other one is an administrative role in a Chinese company located in the urban city center.

Mey demonstrates remarkable fluency in both English and German. Their adolescent daughter attends a Gymnasium at a nearby high school, where she receives weekly lessons in Chinese Mandarin, swimming, judo, stage performance, and piano. Given Den’s enthusiasm for sports, their daughter actively participates in various athletic endeavors. The family resides in a semi-detached house nestled within a tranquil suburb of the metropolitan area. Mey’s trajectory does not conform to other participants. In a unique manner, she embodies the archetype of a self-determined migrant, finding herself in a foreign land without the customary support of familial or communal bonds, and lacking the privilege of substantial economic or cultural resources to bolster her prospects. While the absence of institutionalized support and a dearth of advanced educational qualifications may put constraints on her, Mey’s exceptional attributes lie in her proficient fluency in German, English, and Mandarin, as well as her relentless involvement in social networking.

### 5.5 Lin’s family

I encountered Lin approximately 1 year ago while visiting a restaurant where she was employed as a waitress. Our interaction was limited to the customer relationship, and I observed her to be rather reserved. However, she willingly agreed to participate into this study. Lin originates from a financially disadvantaged peasant family in a rural region of China. After completing her high school education, she migrated to the Guangdong province as an internal migrant and secured employment at a Taiwanese-owned electrical factory. Subsequently, Lin’s sister’s marriage to an Italian-Chinese man presented an opportunity for her to join her sister in Italy through their brother-in-law’s kinship. Upon arrival, she found employment in a garment factory owned by an individual from her brother-in-law’s hometown. Initially, her role involved cooking for the 16 Chinese workers employed in the factory. After 8 months, she transitioned to another factory where she not only engaged in cooking but was also permitted to assist with the garment production process, which entailed paid work, unlike the cooking duties. It was during this period that she met her husband, an Italian, leading to their marriage and the birth of their two children. Additionally, Lin established a store specializing in clothing and shoes sourced from her home province, Fujian, in China.

In 2019, Lin divorced her husband and moved to Germany with her two children. Initially, she found employment in a Chinese restaurant but unfortunately lost her job during the Covid-19 lockdown. Following the lifting of restrictions, Lin’s niece, who resided in the big city, assisted her in securing another position. Lin and her daughter relocated from Berlin to the big city, while her young adult son returned to Italy. Throughout the duration of the fieldwork, Lin resided in a one-bedroom apartment with other Chinese kitchen staff from the restaurant. The main bedroom was occupied by the chef, while the staff members lived in the living room. Lin herself resided in the kitchen area. Her daughter lived with Lin’s niece and her niece’s family at another location in the city, and Lin would visit her daughter every 2 weeks. Lin’s migratory trajectory distinctly aligns with labor migrant groups originating from Fujian, Zhejiang, and Guangdong provinces, characterized by the presence of supportive village and kin ties in Germany. These ties facilitated the acquisition of her current employment and provided support for child-rearing, enabling her to work in the restaurant.

### 5.6 Transnational intergenerational relations – the grandparents in China

The transnational interactions in the family sphere constitute a part of their social contact during their daily activities and are usually oriented by the children. However, the participating families do not visit China as often as imagined, since some of the children are at school-age, and the COVID-19 pandemic also made international traveling more difficult, particularly trips to China. At the end of the fieldwork, Bey and Lin both made a 3-week trip back to China. Bey’s purpose was to visit her elderly parents who were at their eighties as well as to put her Shanghai apartment back on the rental market, while Lin wanted to visit her very sick mother. Lin’s daughter was left to stay with her niece’s family, and Bey’s children were left home with the German grandma and Fin. During my visits to these two families, I observed minimal online communication between the children and their grandparents in China. Lin indicated that her children had no contact with her parents, while Bey described the interactions between her family in Germany and her family of origin in China as sparse:

Lin: “No contact, they grew up here and my parents have so many children and grandchildren in the countryside in China, you know, and they are old and sick, cannot travel, and do not even speak Mandarin.”

Bey: “I will not go back to China to visit them with the children often. My parents are too old, they cannot find any topics to chat with the children. Normally, when we do WeChat they would greet the children and then they are silent. There is also a language problem. Both boys are learning Mandarin, my parents speak Zhejiangnese. The children cannot understand what my parents said once the conversations get longer. Once per year, we four will fly to Hainan for a nice beach holiday. If they want to join us, they can.”

Online communication with grandparents and the challenges in nurturing emotional bonds with grandparents were mentioned by different participatory families in this study. Intergenerational relationships are shaped not only by life stages, health issues, and language barriers but also by the constraints of travel mobility. For instance, during WeChat video calls with her mother, I observed that the interactions between Mey’s daughter and her grandmother were quite brief. Mey’s daughter would typically greet her grandmother in Mandarin, who would reply in Mandarin before switching to the Hunan dialect. Often, the grandmother would continue speaking in Hunanese, leading the grandchild to say goodbye and leave the room. When I inquired about this, Mey explained:

“What can she (Mey’s daughter) talk to them? My father had nothing to talk to small children and my mother cannot understand Mandarin (Mey’s mother only speaks a type of Hunanese). Plus, she is a teenager, probably (she) has no interest in talking to her grandparents in China. And really, they have not seen each other for too long. The relationship between them needs not to be pledged. Too far, we are too far away.”

Resonating with Bey, Mey stressed the long-lasting geographic separation and language bias that made her parents and her daughter’s online communication difficult. In addition, the interview concerns over adolescence and its influence on transnational intergenerational communication. When asked if her parents will come over to Germany to visit them, Mey said:

“Forget it, they are farmers, they would have got lost the moment they left the hometown. Traveling abroad and English?! Even when they traveled in China, my brother always needed to accompany them. Forget it. No way.”

Recent studies have emphasized the limited traveling mobility of Chinese rural farmers (Huang et al., 2024; Zhao and Yu, 2020, 2021). Data from both Lin’s and Mey’s families reveal that the rural backgrounds and low mobility levels of Chinese mothers’ families in China significantly impact the fragile nature of transnational grandparent-grandchild relationships in the intercultural families. Additionally, the age gap between generations and language barriers emerge as key challenges in establishing and maintaining these grandparent-grandchild connections across all three families. If the former is an unavoidable aspect of life, my question about the latter is why all the mothers of these three families insist on having their children learn only Mandarin while in Germany. This question will be explored in more depth in the next section, which examines how transcultural families navigate their experiences through Mandarin language acquisition and the underlying language ideologies driving this choice.

Having identified the difficulties in transnational intergenerational communication in Bey’s, Lin’s, and Mey’s families, my observation of Wey’s family draws a different picture. As illustrated above, Wey is from a prosperous Shanghai family whose parents are in their late fifties and early sixties. During the time of the fieldwork, Wey’s mother came to Germany to visit her and help the couple with child-rearing. Wey recalls the whole family transnational communication plan:

“My mom is here for three months as it is the longest period for a Chinese national to stay in Germany on this traveling visa, then my father will come over for the next three months. Then mom will come over again. They said they will do this in turn till the child goes to school. My father can manage his business virtually.”

During my visit to Wey’s family, I witnessed frequent online communication between the grandma, the toddler, and the grandpa. Sometimes, the conversation with the child would last up to more than an hour. It is also noticeable that whereas in the other three families, the husbands had hardly transnational communication with their in-laws in China, with Wey’s family, the husband Ben was very engaged in communicating with his in-laws in Shanghai. This may be caused by his capability to speak some Shanghainese and Mandarin.

The aforementioned data reveals the contrasting nature of transnational intergenerational communication. On the one hand, life stage and age exert significant influence on transnational family communication. On the other hand, socio-economic and cultural background, along with linguistic capability, wield considerable impact on the divergent patterns of transnational family communication observed within the participating families. For instance, the significant age gap between the grandparents and grandchildren in Lin and Bey’s families has hindered intergenerational transnational communication, while data from Families Lin and Mey emphasize the crucial role of the grandparents’ mobility in their relationship with their grandchildren overseas. Additionally, in all three families, language barriers further complicate transnational communication, as the grandparents lack proficiency in Mandarin, and the grandchildren, raised in Germany, are not fluent in their grandparents’ native language. This linguistic mismatch engenders challenges in effective communication between the generations. Conversely, Wey’s family presents a contrasting scenario, as the grandparents remain relatively youthful and possess the means, both financial and practical, to commute between China and Germany. Consequently, their transnational communication appears notably more fluid. Wey’s family presents a distinct scenario that aligns with the findings of previous studies on the involvement of grandparents in childcare within Chinese communities in France, Britain, and the United States (Wang and Schwartz, 2016; Lie, 2010; Xie and Xia, 2011). The data from Wey’s family supports the conclusions drawn in those studies. Thus, when the involvement of grandparents in childcare is taken into account, it is crucial to consider factors such as their financial status and age, as they can significantly impact the dynamics and outcomes of transnational grandparental caregiving. Notably, Ben, Wey’s husband, exhibits proficiency in Mandarin and even demonstrates a command of the Shanghainese dialect, enabling him to engage in frequent and meaningful communication with his in-laws, both virtually and in person, as observed throughout the field notes. This seems to suggest that the German spouse’s linguistic fluency serves as a catalyst for a more harmonious and fluid transnational communication within the family unit.

### 5.7 Transcultural family upbringing through mandarin learning

Despite some difficulties in maintaining emotional bonds between the children in Germany and their grandparents in China, there is an overt emphasis on Chinese culture and Mandarin learning in the intercultural family upbringing practice. For instance, I had the opportunity to meet Lin’s daughter, who spoke Mandarin fluently. When I inquired about the reasons behind her daughter’s Mandarin proficiency, Lin shared:

“I taught them, I taught them 5 sentences per day, and we started very early. I also taught them how to write and read, 5 words per day since primary school.”

In response to further questioning about the importance of learning Mandarin and her decision to personally teach the children, Lin replied:

“We lived in a suburb, quite far from the Chinese school in the city in Italy. The children had time after school, and I, as the owner of a small shop, had the flexibility to accommodate their learning schedule. It was such a delightful time with my little ones. After school, the three of us would sit together in my small shop in Italy and learn Chinese. I would often laugh at their earnest efforts to learn the language. I brought primary textbooks from China and taught them myself. My husband at that time supported us too, and sometimes he would come to the shop to visit us during our ‘Chinese lessons’. You know, he never really liked the shop…They (the children) learned so quickly. My son can now read Chinese novels, and my daughter only communicates with me in Mandarin on WeChat. Learning Chinese is important, even the foreigners are starting to learn the language. Once the children express a desire to visit China, they can navigate themselves. Learning Chinese is like acquiring an additional language, along with two or three foreign languages.”

Despite the Chinese language proficiency of her children, Lin’s family maintains a primary focus on the joy of learning together as ‘transcultural family doing’. What sets them apart is the significant emphasis placed on Chinese language acquisition within a small Chinese shop in Italy. This assemblage introduces a unique element of the transcultural upbringing. Lin’s depiction of the Mandarin language learning, the Chinese shop, her ex-husband’s support by visiting the shop and the host country of Italy suggests a transcultural and transnational orientation where ‘different trajectories of people, semiotic resources and objects meet at particular moments and places’ (Pennycook, 2019, p. 86) to create a meaning-making process in ‘doing the transcultural family’. By engaging with the mother’s national language within the context of an Italian setting, Lin’s family not only exemplifies a transcultural orientation, bridging the gap between Chineseness and the host country’s culture but also the development of a new transcultural family identity during the lessons, in which all members of the intercultural family participated.

**Figure 1:**
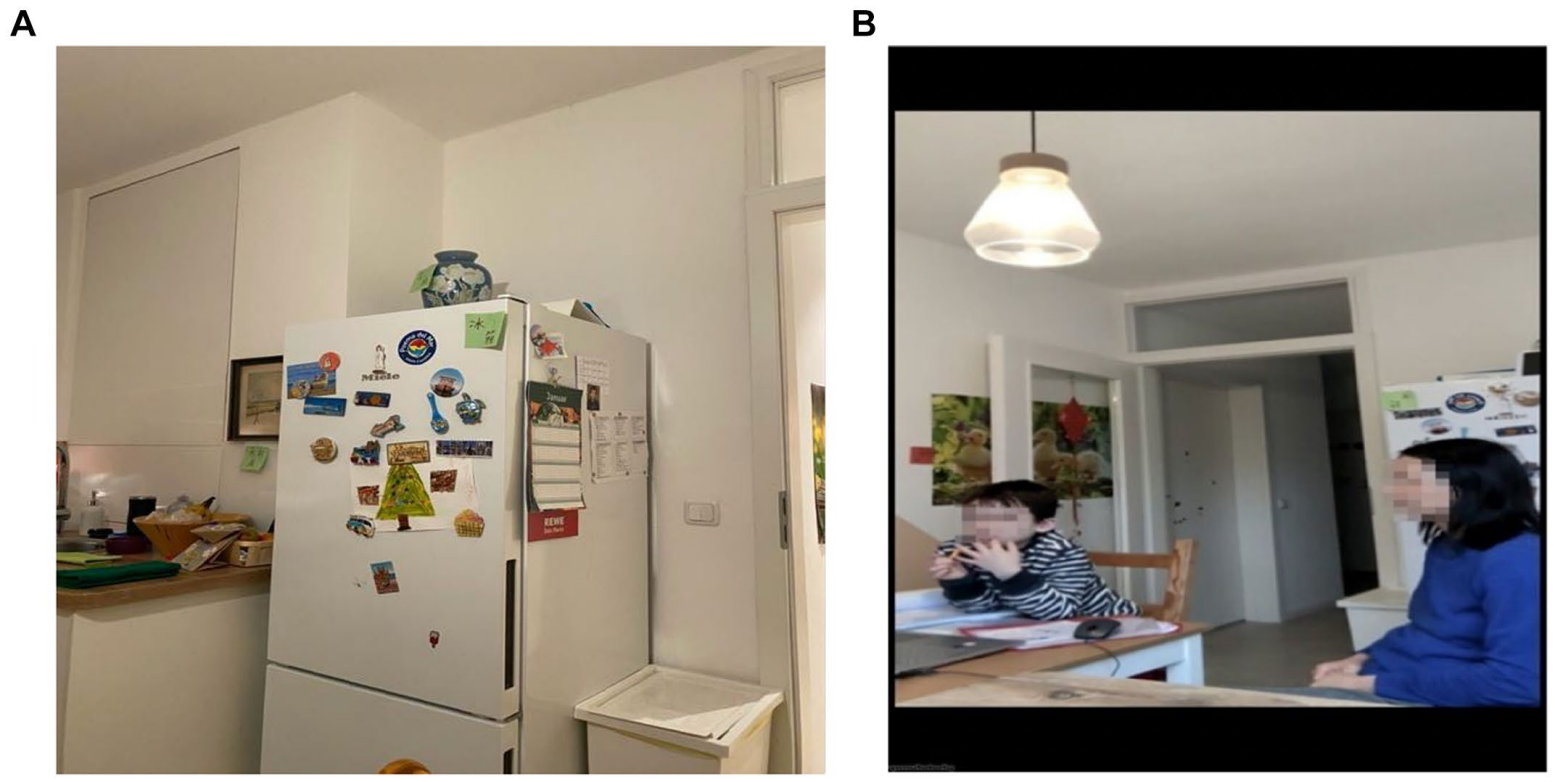
‘Semiotic assemblage’ in the kitchen and transnational Mandarin Learning.

During the fieldwork, Bey frequently discussed her older son’s Mandarin language learning journey. She expressed her concerns, worries, frustrations, and satisfactions regarding her sons’ progress in learning Mandarin. The acquisition of the Mandarin language had become a ritual within Bey’s family, permeating their daily lives and activities. In the image on the left below (Figure 1A), we observe the presence of Mandarin characters prominently displayed in Bey’s family kitchen, symbolizing the ‘semiotic assemblage’ (Pennycook, 2019) of the language’s significance and integration into their home environment. Bey explained that she prepared all the pictures, while Fin artfully arranged and hung them throughout the kitchen. Additionally, the image on the right below (Figure 1B) portrays the transnational nature of their Mandarin learning process, which takes place online.

The location of the photos taken is in a household in Germany, the focus of the on-line learning is Chinese Mandarin language, the participants are the Chinese mother and the Chinese-German son while the Chinese teacher was in her home in Spain. In the background, the Chinese characters gracing the wall and fridge in the kitchen were created by the Chinese mother and hung by the German father. Following the notion of ‘spatial repertoire’ (Canagarajah, 2017), the data of Bey’s family suggests that the time zone is a more salient factor for the transnational learning whereas various spatial locations were used to support the learning process and the construction of transcultural family making took place in a particular physical locality in Germany.

In a different approach, Mey and her husband opted to enroll their child in a Chinese school which has served as a Chinese learning hub for many Chinese-German children in Germany (Li-Gottwald, 2022). Although the learning journey has presented its challenges, with the unwavering support of her parents, Mey’s daughter had been attending the Chinese school for almost 8 years at the time of the fieldwork. Every Sunday, during the school semesters, the entire family undertakes a 45-min drive from their hometown to the nearby city to ensure their daughter’s attendance at the Chinese school. When I inquired about the potential exhaustion stemming from this routine, the German husband responded to me, highlighting the commitment and dedication they have toward their daughter’s Chinese language education:

“It is not exhausting at all. I get up anyway very early. It is easy to drive on Sunday and afterwards we will go to a Chinese restaurant, like a Sunday family outing.”

The wife, Mey later adds:

“It is important to let her to go to the Chinese school so that she has some Chinese culture touch, it is also interesting for me to be embraced by Chinese people, exchange with Chinese or mixed (intercultural) parents and enjoy Chinese cultural activities there.”

The data above indicates that in Mey’s family, their focus on learning the Chinese language extends beyond mere linguistic proficiency. It encompasses the entire learning process as well as the family activities associated with it. The involvement of all three members of the family, the inclusion of a family Sunday outing, the enjoyment of delightful Chinese cuisine, the active engagement in Chinese cultural activities, and interactions with other intercultural parents in a German city presented in the interviews with the couple, serve as a tangible manifestation of their ‘transcultural family doing’. This aspect of their upbringing, as partly documented in Li-Gottwald’s (2023) study on parental social interactions at a Chinese complementary school, highlights the significance of these activities associated with Chinese learning in fostering a sense of transcultural family identity for the intercultural Family May in Germany.

The observational data collected from the Wey family illustrates a picture of transcultural family language practices in which multiple languages are engaged. Wey typically speaks to her daughter in Mandarin, the grandparents use Shanghainese, and her German husband communicates in German. However, there is a significant amount of family interaction characterized by translanguaging (Canagarajah, 2013; García, 2009; García and Li, 2014), where multiple languages are used simultaneously. In the interview, Wey emphasizes:

“It is very important that we speak different languages to Carol (our daughter). Sometimes we mix languages, and that’s fine. It’s our family, and we are very open-minded. The most important thing is that Carol grows up in a multilingual environment. Besides Mandarin and German, Shanghainese is also crucial so she can communicate well with my parents, my mother’s Mandarin is rather limited, and my grandparents are also very happy that Carol can understand their Shanghainese. And you (the researcher), should speak to her in English in the future. Hahaha.”

During the interview, Wey’s husband, who was playfully interacting with their child on the floor, chimed in with a playful remark in Mandarin:

“Our family is big and everywhere, we speak all languages.”

By the end of the fieldwork, Wey had enrolled their two-year-old daughter in a singing class at a Chinese school.

Observations and interviews with the Wey family suggest that the translanguaging practice (Creese and Blackledge, 2010; Li and Wu, 2009) with the daughter and within the family is one of their approaches to their intercultural family life (including the grandparents). By seamlessly blending Mandarin, Shanghainese, and German in their daily interactions, they foster an environment where multiple languages coexist and enrich their daughter’s upbringing. This multilingual practice not only allows for effective communication across three generations but also promotes a new family identity as a cosmopolitan transcultural family, embracing and embodying the fluidity and interconnectedness of modern global cultures (Canagarajah, 2012). As shown in the interview and field notes, it is important to note that Wey’s husband plays an active role in co-constructing their transcultural cosmopolitan family system, emphasizing both the size of the family and its global scope. Additionally, Wey emphasized the importance of her daughter learning Shanghainese, along with other languages, for intergenerational communication and future relationships.

We observe various strategies in transnational family dynamics through the context of Mandarin language learning. Although Bey’s family places a strong emphasis on language proficiency, the process of actively supporting their children’s Mandarin education is central to developing their transcultural family identity. This involves a blend of objects, pictures, online learning across distances, and engaged parental participation. For Lin’s and Mey’s Families, learning Mandarin becomes a collective ‘doing transcultural family’ endeavor. In these families, the process of learning Mandarin transcends individual achievement and instead becomes a shared activity that involves the entire family unit. Therefore, Mandarin language learning serves as a way to engage in family activities and strengthen intercultural familial bonds. Wey’s family takes a different path in constructing a cosmopolitan transcultural family identity through using multiple linguistic resources. However, it is noteworthy that each family adopts a distinct approach to non-Mandarin Chinese child-rearing. While the Wey family places strong emphasis on nurturing their child in their hometown language to foster close intergenerational relationships with the mother’s family in Shanghai, the other three families minimize the importance of learning their hometown languages. Instead, they emphasize the socio-cultural values embedded in the national language. These contrasting scenarios reflect the concept of family configuration as outlined by Widmer (2016), where families are process-oriented networks of functional interdependencies and individuals cooperate as well as sometimes hinder each other, both voluntarily and involuntarily. Thus, the fulfillment of needs such as emotional support, financial and practical resources, and social recognition is mutual within families (Widmer, 2016, p. 61). This significant difference between Wey’s family and the other families partly explains the close intergenerational relationships in Wey’s family compared to the relatively loose grandparents/children’s relationships in the other three families.

## 6 Conclusion

The article illustrates the dynamics of transnational intergenerational relationships and transcultural family upbringing through Mandarin language education within four intercultural families, exploring how some of the discourses on these two topics are interrelated. Although numerous previous transnational family studies reveal the frequent online communication with grandparents in nurturing intergenerational family emotional bonds. The current study reveals that establishing stable and enduring transnational grandparent-grandchild relationships necessitates consideration of factors such as age, generational life stages, grandparents’ travel mobility, languages, and financial resources. A stable transnational grandparenting in intercultural families is heavily reliant on the availability of resources, the constellation of various variables and the interdependency of all family members (Widmer, 2016, 2021). Thus, the four intercultural families exhibit diverse dynamics in their transnational grandparent-grandchild relationships, with a notable challenge of the absence of a shared heritage language. While all four families emphasize the importance of learning Mandarin, only one family also highlights the value of learning both Mandarin and a local Chinese dialect, such as Shanghainese, to enhance transnational communication with grandparents. This particular family, which engages in the most intensive transnational parenting, aligns with Widmer’s (2021) concept of family figuration, where family relationships are shaped by various social, economic, and cultural factors, as well as the consequences of each member’s actions.

Interestingly, although all families prioritize Mandarin, the support process for language learning has established a ‘third space’ (Bhabha, 1990, 1994; Fahlander, 2008)/ the ‘thirdness’ (Zhou and Pilcher, 2019) that fosters a transcultural family dynamic (Crippen and Brew, 2007, 2014). Some families construct their transcultural family identity through intercultural and language learning activities, others through translinguistic practice, and some through the assemblage of objects and active parental engagement.

It is important to note that these four families do not represent the entirety of intercultural families in Germany. Ethnographic research is primarily concerned with gaining a profound understanding of the real-life experiences of individuals or groups within their cultural and social milieu. It is worth noting that this article primarily focuses on data related to the theme of Chinese dimensions in the intercultural families. This emphasis is a result of both space constraints and the desire to provide a comprehensive and in-depth examination of this specific aspect. Other themes, such as German dimensions, will be explored in forthcoming articles, allowing for a more comprehensive analysis of the broader context.

## Data Availability

The original contributions presented in the study are included in the article/supplementary material, further inquiries can be directed to the corresponding author.

## Footnotes

^1^In this paper, the concept of ‘migrants’ is based on the IOM’s (International Organisation for Migration) definition for migration: ‘movement of a person or a group of persons, either across an international border (international migration), or within a state (internal migration), encompassing any kind of movement of people, whatever its length, composition and causes’. It includes migration of refugees, displaced persons, economic migrants and persons moving for other purposes, including family reunification. https://home-affairs.ec.europa.eu/networks/european-migration-network-emn/emn-asylum-and-migration-glossary/glossary/migration_en#:~:text=Thus%20it%20includes%20migration%20of,other%20purposes%2C%20including%20family%20reunification.

^2^Pseudonym as requested by the participant families.

^3^Pseudonym as requested by the participant family.
